# Molecular hydrogen is comparable to sulfasalazine as a treatment for DSS-induced colitis in mice

**DOI:** 10.17179/excli2021-3762

**Published:** 2021-06-29

**Authors:** Tyler W. LeBaron, Fereshteh Asgharzadeh, Majid Khazaei, Branislav Kura, Alex Tarnava, Jan Slezak

**Affiliations:** 1 Centre of Experimental Medicine, Institute for Heart Research, Slovak Academy of Sciences, Faculty of Natural Sciences of Comenius University, 841 04 Bratislava, Slovak Republic; 2 Molecular Hydrogen Institute, Utah, USA; 3 Department of Kinesiology and Outdoor Recreation, Southern Utah University, Cedar City, 84720, Utah, USA; 4 Department of Physiology, Faculty of Medicine, Mashhad University of Medical Sciences, Mashhad, Iran; 5 Metabolic Syndrome Research Center, Mashhad University of Medical Sciences, Mashhad, Iran; 6 Drink HRW and Natural Wellness Now Health Products Inc., Unit C 60, Braid St, New Westminster, BC, Canada

**Keywords:** molecular hydrogen, colitis, inflammation, sulfasalazine, oxidative stress

## Abstract

Colitis is an inflammatory condition of the bowels associated with abdominal pain, diarrhea, fatigue, and fever. Its etiology is multifactorial but related to the overproduction of inflammatory and oxidative mediators. There is currently no cure for this disease, and drugs used to manage it often have deleterious side effects. H_2_ is recognized as having anti-inflammatory and antioxidant effects, which may qualify it as a novel therapeutic for colitis. We induced an acute model of colitis in mice by administering dextran sulfate sodium (DSS) in drinking water for seven days. Mice were divided into five groups (n=6); normal, colitis, H_2_-treated colitis, sulfasalazine-treated colitis, and H_2_ plus sulfasalazine-treated colitis. From days three to ten, mice were given H_2_, sulfasalazine, or both. H_2_ was administered via dissolving a hydrogen-generating tablet in water to make hydrogen-rich water (HRW), which was ingested ad libitum and via oral gavage (200 μL). The Disease Activity Index (DAI), histological changes, and markers of inflammation and oxidative stress were assessed. HRW and sulfasalazine significantly improved bodyweight, DAI, mucosal damage, crypt loss, and spleen weight compared to control. Both treatments significantly decreased inflammation (high-sensitive C-reactive protein) and restored redox balance (total thiol, superoxide dismutase, catalase activity). There was a trend for the combination treatment to be more effective than either HRW or sulfasalazine alone. Furthermore, HRW tended to be as effective as, and often more effective than, sulfasalazine. HRW may serve as a therapeutic for ameliorating DSS-induced colitis in mice.

## Introduction

Idiopathic inflammatory bowel diseases are associated with a high burden of morbidity and progressive disability (Piovani et al., 2019[22]). Colitis is one of the principal forms of inflammatory bowel disease and there is no effective treatment or cure (Piovani et al., 2019[22]). The annual costs associated with its management are estimated to be as high as nearly $ 15 billion in the USA alone (Gajendran et al., 2019[6]). The presumed etiology of colitis is considered to be multifactorial involving genetics, autoimmunity, the microbiome, and various environmental factors (Gajendran et al., 2019[6]; Piovani et al., 2019[22]). These contribute to increased inflammation and excessive production of reactive oxygen species, both of which further induce and exacerbate colitis (Gajendran et al., 2019[6]; Piovani et al., 2019[22]). 

Molecular hydrogen (H_2_ gas) has recently been recognized as a novel medical therapeutic with anti-inflammatory, antioxidant, and signal modulating effects (LeBaron et al., 2019[14]). Molecular hydrogen is a stable diatomic gas and can be administered either via inhalation or ingestion of water that contains dissolved H2, hydrogen-rich water (HRW) (LeBaron et al., 2019[14]). Clinical studies have demonstrated its benefits in many different areas including exercise medicine (LeBaron et al., 2019[15][16]), cognitive impairments (Nishimaki et al., 2018[20]), stroke (Ono et al., 2017[21]), cancer (Akagi and Baba, 2019[1]), metabolic syndrome (LeBaron et al., 2020[17]), and recently in patients with COVID-19 (Guan et al., 2020[7]; Russell et al., 2020[23]). 

HRW has been previously studied in an animal model of inflammatory bowel disease with favorable effects (Kajiya et al., 2009[12]). However, it is unclear how effective HRW is compared to standard drug treatment such as sulfasalazine. Additionally, we used drinking HRW and oral gavage instead of H_2_-rich saline, and a higher concentration of HRW. Accordingly, we induced an acute model of colitis induced by dextran sodium sulfate (DSS) and compared and contrasted the antioxidant and anti-inflammatory effects of HRW with the drug sulfasalazine. 

## Materials and Methods

### Drugs and chemicals

High-concentration HRW was made via hydrogen-producing tablets (HRW Natural Health Products Inc., New Westminster BC, Canada). For drinking, HRW was prepared twice daily every 12 h by dissolving one tablet in a sealed 500-mL soda bottle with no headspace. The initial concentration of hydrogen water was > 1.5 mM and remained > 0.1 mM by end of 12 h as measured by redox titration (H_2_Blue; H_2_Sciences, Las Vegas, Nevada). Dextran sodium sulfate (DSS‐40kD), Hematoxylin and Eosin (H&E), and malondialdehyde (MDA), total thiol, superoxide dismutase (SOD), and catalase materials were all purchased from Sigma (Sigma Chemical Co., USA). The colitis drug sulfasalazine was also obtained from Cayman Co.

### Ethics statement

Thirty C57BL/6 male mice (6-8 weeks old) were provided by the Pasteur Institute of Iran (Tehran, Iran) and maintained according to the standard protocol of the Guidelines on Institutional Animal Care from Mashhad University of Medical Sciences. Mice received water and food *ad libitum* and were kept in an air-conditioned room with a laboratory temperature of (22-25 °C), 12 hr light/dark cycle.

### Murine colitis model and experimental protocol

As illustrated in Table 1(Tab. 1) mice were randomly divided into five groups (n = 6 for each group): i) the control group, which received drinking water for the full 10 days, ii) the colitis group, which received 1 % (w/v) DSS in drinking water for 7 days (≈ 3.3 mL/mouse/ day) followed by normal drinking water for the next 3 days, iii) the sulfasalazine-treated group, which received 1 % (w/v) DSS in drinking water (≈ 3.3 mL/mouse/day) from day 1-7, and 100 mg/kg/day of sulfasalazine from day 3-10 via oral gavage, iv) the HRW-treated group, which received DSS 1 % (w/v) in drinking water (≈ 3.3 mL/mouse/day) from day 1-7, and HRW from day 3-10 both from drinking and 200 μL (> 1.5 mM H_2_) via daily oral gavage, and v) the combination-treated group, which received 1 % (w/v) DSS in drinking water (≈ 3.3 mL/mouse/day) from day 3-7, and 100 mg/kg/day sulfasalazine and HRW via drinking (ad libitum) and via oral gavage (200 μL) from day 3-10. 

### Assessment of colitis

During the study, weight change, stool characteristics, rectal bleeding and rectal prolapse were reported daily. The disease activity index (DAI) data was given as an average body weight change score, stool consistency, and rectal bleeding and prolapse as previously described (see Table 2(Tab. 2)) (Cooper et al., 1993[5]). 

### Histopathological evaluation of colons

At the end of the experiments, mice were euthanized, and the colon was removed, washed, and its weight and length were measured. The formalin-fixed colon tissues were washed, paraffin-embedded, sectioned, stained with hematoxylin-eosin (H&E) and Masson's trichrome, examined by light microscopy, and graded according to the standard histopathological criteria provided in Table 3(Tab. 3).

### Tissue preparation to measure oxidative stress markers

The colon tissues were weighed and homogenized in ice with PBS. Then, at 4 ° C, the homogenate was centrifuged for 20 min at 10000 rpm. The supernatant was collected and stored at - 70 °C for evaluation of oxidative/antioxidative markers including malondialdehyde (MDA), total thiol, and superoxide dismutase (SOD) and catalase activity (Chassaing et al., 2014[4]).

#### MDA measurement

Malondialdehyde (MDA) was measured by mixing 1 mL of 10 percent homogenate with 2 mL of a solution containing thiobarbituric acid, trichloroacetic acid, and HCL in boiling water for 45 minutes and centrifuged for 10 minutes. The absorbance was read at 535 nm and the MDA levels were calculated using the formula C(M) = (A/1.65 = 105). 

#### Total thiol group measurement 

Total thiol concentration was calculated using the di-thio nitrobenzoic acid (DTNB) reagent. 1 mL of Tris-EDTA buffer (pH=8.6) was added to the colon homogenate. Test absorbance was read at 412 nm against the Tris-EDTA buffer alone (A1). This solution was applied to 20 μL of DTNB reagents and kept at room temperature for 15 minutes. Next, the sample absorbance was reported again (A2). DTNB reagent absorbance was reported alone as a blank (B). The total concentration of thiol (mM) was determined using the following formula: (mM) = (A2 − A1 − B) × (1.07/0.05 × 13.6).

#### Determination of SOD activity

SOD activity was measured using a colorimetric assay described by Madesh and Balasurbamanian (1997). The method is based on the synthesis of superoxide dismutase by pyrogallol auto-oxidation and inhibition of superoxide-dependent reduction of 3-(4,5-dimethyl-thiazol-2-yl) 2,5-diphenyl tetrazolium bromide (MTT) to its formazan. The reaction ends with the addition of dimethyl sulfoxide (DMSO), stabilizing the color. In brief, the homogenized colon was poured into the wells and incubated for 5 min at room temperature. The reaction was stopped by DMSO and then read as a reference wavelength with a microplate reader at a wavelength between 570 nm and 630 nm. One unit of SOD is known as the amount of protein required to inhibit a 50 % reduction in MTT (Madesh and Balasubramanian, 1997[19]).

#### Determination of CAT activity

Catalase activity was determined by measuring the rate of H_2_O_2_ hydrolysis at 240 nm in sodium phosphate buffer. The velocity of the enzyme response can be determined by converting H_2_O_2_ to H_2_O and O_2_ within 1 minute of the normal condition (Madesh and Balasubramanian, 1997[19]).

#### Determination of hsCRP

The inflammatory marker, high-sensitive C-reactive protein (hsCRP) was determined using the assay based on latex-enhanced turbidimetric immunoassay. The agglutination of the anti-CRP antibody is detected as an absorbance change (500 nm). The magnitude of the change is proportional to the quantity of CRP in the sample, and concentration is interpolated from the prepared standard calibration curve. 

### Statistical analysis

Results are presented as mean values ± standard error of the mean and analyzed following Tukey's multiple comparison tests by Student's t-test or ANOVA. Software analyses were conducted using SPSS v.20 statistical software (IBM, Chicago).

## Results

### H_2_-rich water improved clinical symptoms of colitis

**Figure 1:**
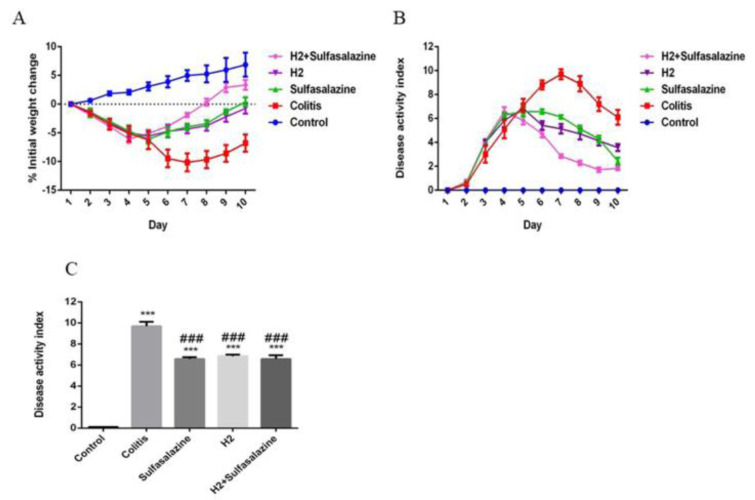
Clinical symptoms of DSS-induced colitis in different groups. (A) The % initial weight change was shown as the average daily weight of days 1 to 10. (B) scores of the Disease Activity Index (DAI) daily. (C) Highest DAI during the experiment. Results are expressed as means ± SEM (n = 6). ***P < 0.001 compared to control and ### P < 0.001 compared to the colitis group.

Animal weight was monitored daily. The body weight of mice in the control group continued to increase, while the body weight of the mice treated with DSS decreased during the experiment. In comparison, mice body weight in the H_2_, sulfasalazine, and combination groups significantly improved after treatment (Figure 1A(Fig. 1)). The DAI scores for the DSS-treated mice were significantly higher compared to the control group (P < 0.001). However, the DAI scores for the sulfasalazine, H_2_, and combination groups were all significantly lower compared to the colitis group (P < 0.001) (Figure 1C(Fig. 1)).

### H_2_ ameliorated colon tissue damage in colitis model

**Figure 2:**
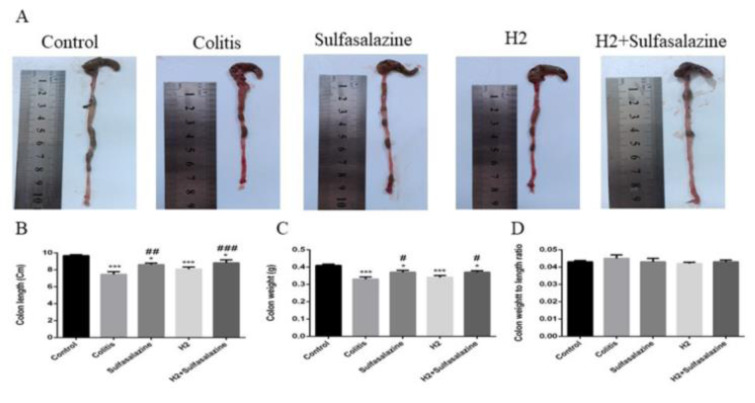
The effects of H2 and sulfasalazine on DSS-induced colon aberrations. Colon length (A, B), colon weight (C), colon weight / length ratio (D). Results are expressed as means ± SEM (n = 6). ***P < 0.001 , *P < 0.05 compared to control and ### P < 0.001, ## P < 0.01 and # P < 0.05 compared to colitis group.

In colitis mice, sulfasalazine (P < 0.01) and sulfasalazine with H_2_ (P < 0.001) significantly suppressed DSS-induced colon shortening (Figure 2A, B(Fig. 2)). Similarly, sulfasalazine with and without H_2_ attenuated the decreased colon weight induced by DSS (P < 0.05). Treatment with H_2_ had a non-statistical tendency to decrease the colon weight to length ratio, reflecting a decrease in inflammation and tissue edema (Figure 2D(Fig. 2)). 

### Histopathological evaluation 

**Figure 3:**
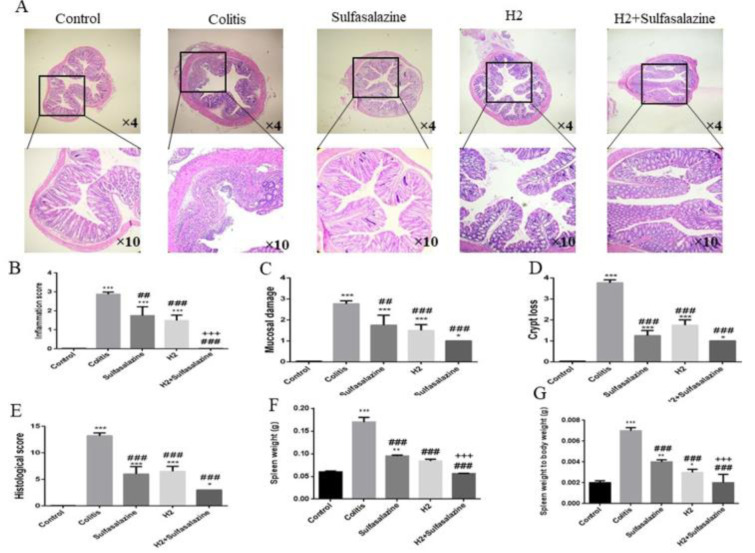
H_2_ has mitigated deteriorating colonial histopathological changes and inflammation in DSS-induced colitis mice. Representative H&E staining images from various treatments of the colonic sections (A), inflammation score (B), mucosal damage score (C), crypt loss (D), histological scores (E), spleen weight (F), and spleen weight to body weight ratio (G) were measured in different groups. Results are expressed as means ± SEM (n = 6). ***P < 0.001 , **P < 0.01 and *P < 0.05 compared to control and ### P < 0.001 and ## P < 0.01 compared to colitis group.

We compared colonic tissue histological damage (Figure 3A(Fig. 3)) between H_2_ treated with and without sulfasalazine to the colitis model and control mice. 

DSS administration resulted in pathological alterations such as inflammation of the mucosa and cell infiltration (Figure 3B(Fig. 3)), destruction of the epithelium cell layer (Figure 3C(Fig. 3)), and crypt loss (Figure 3D(Fig. 3)) resulting in significantly higher histological scores (Figure 3E(Fig. 3)) compared to control group. On the other hand, both H_2_ and sulfasalazine, especially in combination, significantly reduced these histological aberrations in comparison with colitis mice (Figure 3B-E(Fig. 3)). H_2_ was slightly more effective (P < 0.001) than sulfasalazine (P < 0.01) in preventing mucosal damage compared to the colitis group. Furthermore, as expected the spleen weight and spleen weight to body weight ratio were increased by DSS-administration, which is generally associated with the extent of inflammation. However, H_2_ administration both with and without sulfasalazine was able to improve these manifestations in DSS-induced colitis mice (P < 0.001; Figure 3F, G(Fig. 3)).

### H_2_ and sulfasalazine improves inflammation in DSS-induced colitis

The effects of hydrogen treatment alone or in combination with sulfasalazine on high-sensitive C reactive protein (hs-CRP) levels were also evaluated in DSS-induced colitis. 

**Figure 4:**
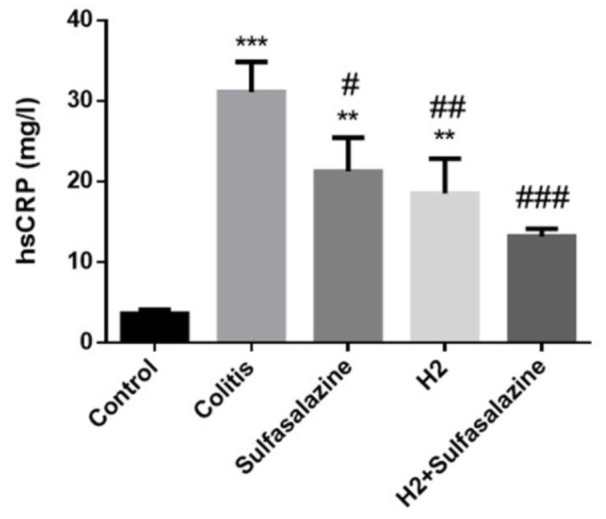
Masson's trichrome staining of colon tissues of treated and untreated colitis mice compared to control (Figure 5A). Digital image analysis of percent collagen content. Results are expressed as means ± SEM (n = 6).***P < 0.001, **P < 0.01 and *P < 0.05 compared to control. ### P < 0.001 and ## P < 0.01 compared to colitis group. +++ P < 0.001, ++ P < 0.01, + P < 0.05 compared to sulfasalazine group.

As illustrated in Figure 4(Fig. 4), DSS treatment significantly elevated hsCRP levels above control in all groups. However, compared to the colitis group, hsCRP was significantly reduced in the hydrogen (P < 0.01), sulfasalazine (P < 0.05), and the combination (P < 0.001) groups. 

### H_2_ and sulfasalazine decreases colon fibrosis in colitis 

**Figure 5:**
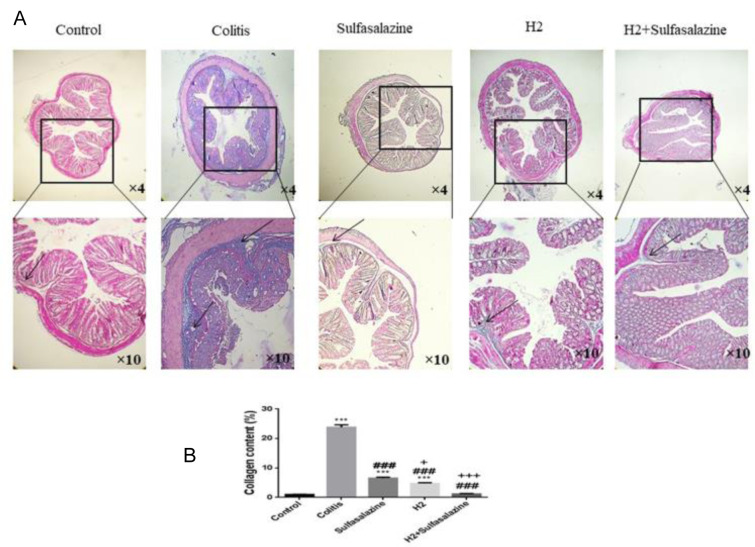
Effects of H_2_ and sulfasalazine on oxidative and antioxidant markers in DSS-induced colitis. Results are expressed as means ± SEM (n = 6). ***P < 0.001 , **P < 0.01 and *P < 0.05 compared to control. ### P < 0.001 and ## P < 0.01 compared to colitis group. +++ P < 0.001, ++ P < 0.01, + P < 0.05 compared to sulfasalazine group.

The colitis group showed more fibrosis in collagen colon tissues of colitis mice as visualized through Masson's trichrome staining (see Figure 5(Fig. 5)). 

H_2_ and sulfasalazine significantly reduced the collagen deposition in the colon induced by DSS (P < 0.001). Compared to sulfasalazine, H_2_ appeared to be more effective statistically (P < 0.05), with maximum effect provided by the H_2_ sulfasalazine combination (P < 0.001) (Figure 5A, B(Fig. 5)). 

### H_2_ and sulfasalazine improves redox status in DSS-induced colitis

**Figure 6:**
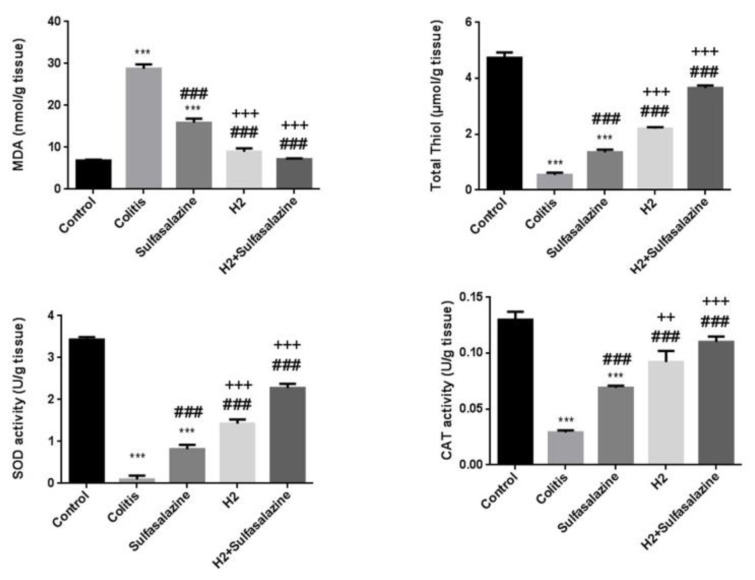
Effects of H_2_ and sulfasalazine on oxidative and antioxidant markers in DSS-induced colitis. Results are expressed as means ± SEM (n = 6). ***P <0.001, **P <0.01 and *P < 0.05 compared to control. ### P <0.001 and ## P < 0.01 compared to colitis group. +++ P<0.001, ++ P<0.01, + P<0.05 compared to sulfasalazine group.

DSS significantly increased oxidative stress and reduced antioxidant status compared to control. However, H_2_ and sulfasalazine either alone or in combination were protective against DSS-induced colitis as illustrated in Figure 6(Fig. 6).

Although sulfasalazine significantly reduced MDA levels compared to the colitis model (P < 0.001), H_2_ with and without sulfasalazine was significantly more effective compared to sulfasalazine treatment (P < 0.001), and essentially prevented the DSS-induced MDA increase. Similarly, although all treatments significantly improved superoxide dismutase (SOD), total thiol, and catalase (CAT) levels compared to colitis (P < 0.001), H_2_ with and without sulfasalazine was significantly more effective (P < 0.001). Additionally, H_2_ with and without sulfasalazine essentially prevented the DSS-induced decline in these antioxidant levels. The combination treatment had a general trend of being more effective, but it was only statistically greater than H_2_ at improving catalase activity levels compared to sulfasalazine (P < 0.01 vs. P < 0.001 for H_2_ and sulfasalazine, respectively). 

## Discussion

In the present study we induced an acute mouse model of colitis using 1 % DSS in drinking water, as has been described previously (Binabaj et al., 2019[3]). Consistent with previous data, we found that DSS led to symptoms of colitis, and that treatment with molecular hydrogen and or sulfasalazine exerted protective effects. From a phenotypic perspective molecular hydrogen was either as effective as, and more effective than, sulfasalazine in improving body weight, attenuating the Disease Activity Index, and in maintaining colon length and weight. Furthermore, this comparative/superior effect was also observed in the histopathological evaluation looking at inflammation, mucosal damage, crypt loss, and spleen weight as well as pathological colon fibrosis. However, the combinational therapy of molecular hydrogen with sulfasalazine was often more effective than when either was administered alone. 

Similar benefits were observed in an acetic acid-induced colitis rat model where H_2_-rich saline was administered once every other day for two weeks (He et al., 2013[8]). They reported reduced weight loss and diarrhea, and less mucosal damage (He et al., 2013[8]). In another report, DSS was used to induce irritable bowel syndrome, which H_2_ treatment also significantly attenuated (Shen et al., 2017[25]). 

On a molecular level, hydrogen has been demonstrated to exert anti-inflammatory and antioxidant effects in animal and human studies (LeBaron et al., 2019[14]). DSS induces excessive inflammation and depletes endogenous antioxidants leading to oxidative stress (Kajiya et al., 2009[12]). Our results show that molecular hydrogen can attenuate inflammation as noted by the reduction of DSS-induced increase in high-sensitivity C-reactive protein (hsCRP). In this case, HRW tended to be slightly more effective than sulfasalazine, but not as effective as the combination. Similarly, HRW prevented the DSS-induced increase in the marker of lipid peroxidation malondialdehyde. This may be due to hydrogen's ability to prevent the DSS-induced decline in the antioxidants, superoxide dismutase and catalase, as well as in the total thiol concentration, as we observed in this study.

These antioxidant effects of hydrogen are in line with previous research, including in humans. For example, ingestion of high-concentration hydrogen water for six months in subjects with metabolic syndrome (n=60) resulted in decreased markers of inflammation and improved antioxidant status in addition to improving metabolic and clinical parameters (LeBaron et al., 2020[17]). One of the mechanisms by which H_2_ exerts its antioxidant effect is via induction of the Nrf2/Keap1 antioxidant system (Kura et al., 2018[13]). The Nrf2 transcription factor regulates the production of over 200 cytoprotective proteins involved in antioxidation and detoxification (Ma, 2013[18]). In an earlier study, the protective effects of hydrogen on DSS-induced colitis were dependent on the induction of heme-oxygenase-1 expression (Shen et al., 2017[25]), which is a downstream target of Nrf2. However, the exact mechanism responsible for H_2_-induced Nrf2 activation remains elusive (LeBaron et al., 2019[14]). 

Interestingly, there is a strong positive relationship between the inflammatory conditions of colitis and rheumatoid arthritis (Attalla et al., 2019[2]). Accordingly, the drug sulfasalazine is a standard drug for both of these inflammatory conditions (Attalla et al., 2019[2]), for which hydrogen has also been shown to be effective. For example, in small clinical studies, treatment with molecular hydrogen has shown pronounced therapeutic effects in patients with rheumatoid arthritis (Ishibashi et al., 2012[10], 2014[11]). Additionally, the drug acarbose, an α-glucosidase inhibitor, has been suggested to be used as a treatment for colitis due its ability to increase hydrogen production from intestinal bacteria (Zhang et al., 2012[29]). 

Dysregulation of the microbiome represents an important factor for numerous pathologies and conditions including colitis (Shen et al., 2018[26]). Although not investigated in our study, several studies suggest that HRW favorably modulates and improves the microbiome in mice (Higashimura et al., 2018[9]; Xiao et al., 2018[28]), goats (Wang et al., 2017[27]), piglets (Zheng et al., 2018[30]), and humans (Sha et al., 2018[24]). Accordingly, HRW may have promoted improved microbiome homeostasis in the DSS-induced colitis mice, contributing to the favorable effects observed in our study. 

In conclusion, our study provides additional evidence that HRW may serve as a potential therapy for patients suffering from colitis, and as a corollary, strongly warrants clinical investigation. Additionally, research into the exact molecular mechanisms of hydrogen, and its influence on the gut microbiome, will help further the understanding and development of this safe and simple therapeutic. 

## Notes

Tyler W. LeBaron and Fereshteh Asgharzadeh contributed equally as first author.

Majid Khazaei and Jan Slezak (Centre of Experimental Medicine, Institute for Heart Research, Slovak Academy of Sciences, Dúbravská cesta 9, 841 04 Bratislava, Slovak Republic; Tel.: +421 903 620 181, E-mail: jan.slezak@savba.sk) contributed equally as corresponding author.

## Acknowledgements

We thank Mr. Alex Tarnava, CEO of HRW Natural Health Products Inc. for kindly donating Drink HRW tablets for this study and thank Mashhad University of Medical Sciences for their support. 

## Conflict of interest

TWL has received travel reimbursement, honoraria, and speaking and consultancy fees from various academic and commercial entities regarding molecular hydrogen. AT is the CEO of HRW Natural Health Products Inc., whose company provided product and additional funds for biomarker measurements. All other authors report no conflict of interest. 

## Funding

This study was partially supported by Slovak Research and Development Agency (APVV)-0241-11, APVV-15-0376; ITMS 26230120009; Scientific grant agency of the Ministry of Education of the Slovak Republic (VEGA) 2/0063/18, and by HRW Natural Health Products Inc.

## Figures and Tables

**Table 1:**
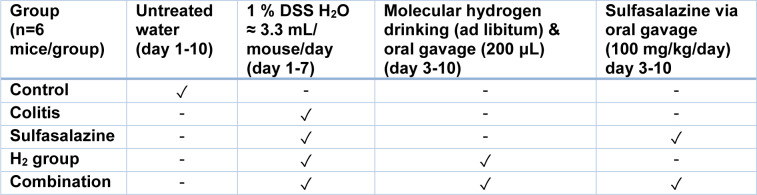
Protocol for inducing and treating experimental colitis in mice

**Table 2:**
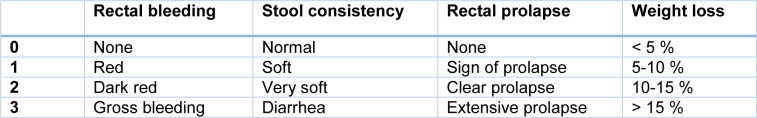
Disease Activity Index (DAI) score system for colitis mice

**Table 3:**
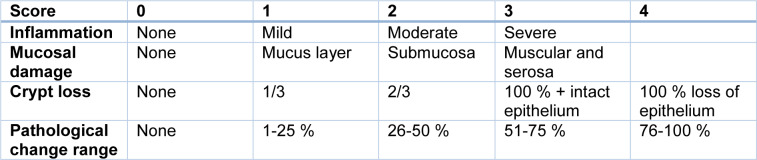
Colorectal tissue damage assessment system based on histopathological criteria
